# USP7 at the Crossroads of Ubiquitin Signaling, Cell Cycle, and Tumorigenesis

**DOI:** 10.3390/molecules30204038

**Published:** 2025-10-10

**Authors:** Matteo Lusardi, Federica Rapetti, Andrea Spallarossa, Marta Massone, Elena Cichero, Chiara Brullo

**Affiliations:** Department of Pharmacy, University of Genoa, Viale Benedetto XV 3, 16132 Genova, Italy; federica.rapetti@unige.it (F.R.); andrea.spallarossa@unige.it (A.S.); massone.marta@gmail.com (M.M.); elena.cichero@unige.it (E.C.)

**Keywords:** Ubiquitin-proteasome system, USP7, deubiquitination, tumorigenesis, USP7 inhibitors, USP7 activators

## Abstract

Protein homeostasis is a dynamic process essential for cellular function and survival, tightly controlled by the ubiquitin–proteasome system. Within this system, ubiquitin-specific protease 7 (USP7) plays a key role as a deubiquitinating enzyme, thus modulating the stability, localization, and activity of a wide variety of substrates. USP7 is involved in critical cellular processes such as DNA repair, apoptosis, immune response, and epigenetic regulation. The dysregulation of USP7 expressions or activity has been linked to several pathological conditions, including cancer, neurodegenerative and inflammatory diseases, and viral infections. This enzyme exerts its biological functions through the stabilization of both oncogenic and tumor suppressor proteins, highlighting its sensitive role in tumorigenesis. Despite the identification of selective USP7 inhibitors with promising preclinical activity, the development of clinically effective compounds remains a major challenge. This review summarizes the current understanding of USP7 structure, function, and biological relevance, with a particular emphasis on its potential as a therapeutic target in oncology.

## 1. Introduction

**Figure 1:**
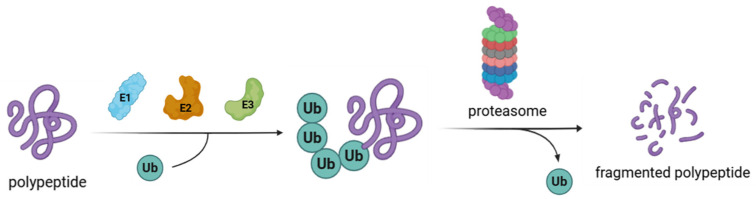
Schematic activity of the ubiquitin proteosome system. Ub = ubiquitin, E1 = E1 ligase, E2 = E2 ligase, E3 = E3 ligase.

Aggregated and/or misfolded proteins are constantly eliminated inside eukaryotic cells to preserve cellular functions. This continuous and complex process, called protein homeostasis, is mainly regulated by the ubiquitin–proteasome system (UPS) (Figure 1) [1,2]. A crucial mechanism in this turnover is represented by protein ubiquitination, a post-translation modification that contributes to cell homeostasis by regulating the stability, localization, and activities of different proteins [3,4,5]. Protein ubiquitination is controlled by deubiquitinating enzymes (DUBs) that selectively remove ubiquitin chains from proteins [6,7]. Among the nine DUB families, ubiquitin-specific proteases (USPs) are the predominant and most studied [8].

Ubiquitin-specific protease 7 (USP7), also known as herpesvirus-associated ubiquitin-specific protease (HAUSP), belongs to the USP family and is involved in the regulation of different pathways such as immune response, epigenetic control, viral replication, cell apoptosis, DNA damage response, and DNA replication and transcription [9,10]. Dysregulations of USP7 expression could lead to different pathological conditions including cancer, viral infections, inflammatory diseases, and neurodegenerative diseases [11,12,13,14]. Consequently, USP7 represents a relevant pharmacological target for the treatment of various pathological disorders; in particular, its aberrant expression in many cancers has been widely studied to explore novel antitumor strategies [15]. In fact, USP7 mediates the deubiquitination and stabilization of oncogenic proteins such as murine double minute 2 (MDM2), murine double minute X (MDMX), and the tumor suppressors p53 [16], phosphatase and tensin homolog (PTEN) [17], nuclear factor-kappa B (NF-κB) [18], β-catenin [19], and forkhead box P3 (FOXP3) [20]. However, despite several USP7 inhibitors having been reported in the scientific literature, the search for potent and selective compounds is still challenging and, unfortunately, to date, no USP7 inhibitors have been approved for clinical use [21].

### 1.1. Structure and Function of the Ubiquitin–Proteasome System

The UPS consists of an intricate and complex network which is mainly implicated in the regulation of ubiquitin conjugation, a multistep process associated with several biological functions including DNA repair, transcriptional regulation, endocytosis, intracellular signaling, and protein trafficking [22].

**Figure 2:**
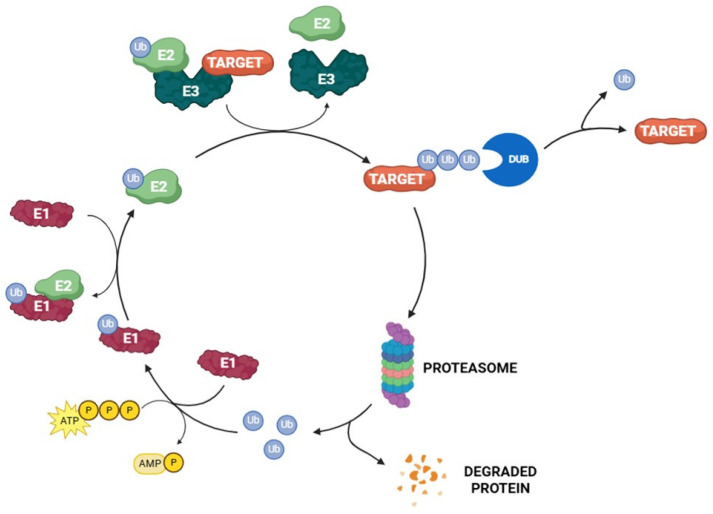
The UPS pathway.

Ubiquitin is a small 76-amino-acid protein, widely found all over eukaryotic cells. Its covalent attachment to the target, mediated by UPS enzymes, regulates protein interaction, localization, and degradation [23]. The ubiquitination process is orchestrated by the sequential action of three different enzymes: E1 (ubiquitin-activating enzyme), E2 (ubiquitin-conjugating enzymes), and E3 (ubiquitin–protein ligase) [24]. The reaction begins with the formation of a thioester intermediate between E1 catalytic cysteine and ubiquitin C-terminal glycine through an ATP-dependent mechanism. In the second step, the activated ubiquitin is relocated to E2 by trans thiolation. Finally, E3 recruits the target protein and transfers ubiquitin from the E2 enzymes to the substrate (Figure 2). Usually, a lysine residue on the substrate attacks the ubiquitin-E2 thioester bond, leading to the acylation of the lysine ɛ-amino group. However, when there are no accessible lysine residues on the target, ubiquitin is conjugated to the substrate N-terminal amino group [25,26,27]. According to the number of ubiquitin moieties attached to the substrate, three different types of ubiquitination linkages have been described. The addition of a single ubiquitin unit on a single lysine residue on the substrate is called monoubiquitination. On the other hand, multi-monoubiquitination occurs when a single ubiquitin unit is conjugated to multiple lysine residues of the target protein. Finally, polyubiquitination refers to the attachment of more than two ubiquitin units to the same lysine residue of the target protein [3,28]. Polyubiquitination can occur on specific ubiquitin lysin residues (namely, K6, K11, K27, K29, K33, K48, and K63) or on the N-terminal methionine M1; these amino acids can be independently ubiquitinated, leading to the formation of different types of ubiquitin chains linked by isopeptide bonds to the substrate protein. K48-Linked polyubiquitin chains are a signal for the 26S proteasome-mediated degradation of the target, whereas the other chains have a non-degradation role [29,30]. 26S proteasome (present in both cytosol and the nucleus of eucaryotic cells) plays a pivotal role in the UPS. In detail, this multi-subunit proteolytic complex degrades a large variety of regulatory damaged and unfolded proteins, acting as a proteome modulator and controlling numerous cellular processes [31].

Protein ubiquitination can be reversed by DUBs, a special class of enzymes associated with 26S proteasome that remove the ubiquitin units from the target protein [32]. Up to now, about 100 DUBs have been identified in humans and are divided into two main categories: cysteine proteases and metalloproteases. According to their sequence and domain architecture, cysteine proteases are further subclassified into eight families, namely ubiquitin-specific proteases (USPs), ubiquitin C-terminal hydrolases (UCHs), Otubain domain ubiquitin-binding proteins (OTUs), Machado–Joseph disease protein domain proteases (MJDs), the MIU-containing novel DUB family (MINDY), monocyte chemotactic protein-induced proteins (MCPIPs), permuted papain fold peptidase of dsDNA viruses and eukaryotes (PPPDEs), and zinc finger with UFM1-specific peptidase domain proteins (ZUFSPs). On the other hand, the class of metalloproteases is only represented by the group of JAMM/MPN domain-associated metallopeptidases (JAMMs) [33,34].

**Figure 3:**
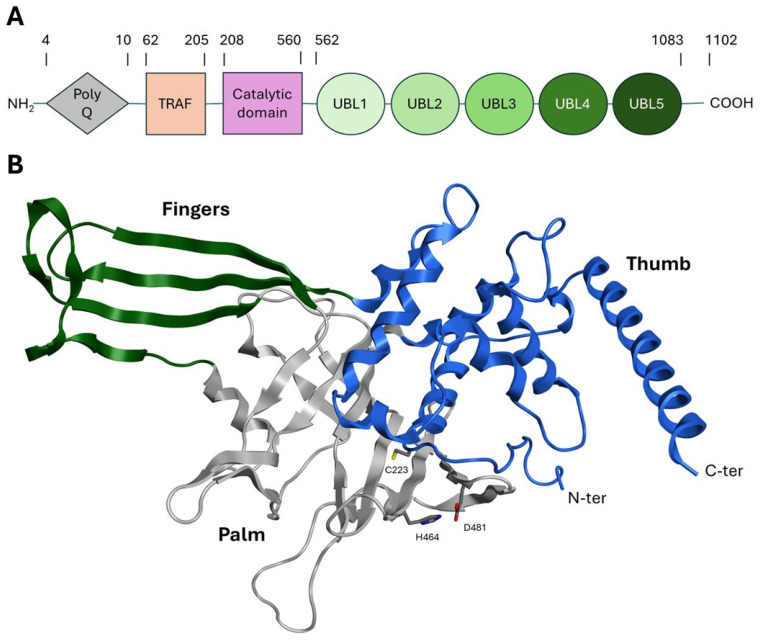
(**A**) USP7 subdomains. (**B**) Crystal structure of the catalytic domain of USP7 (PDB: 59NT). The finger, thumb, and palm subdomains are colored green, blue, and gray, respectively. Catalytic residues are represented as stick models.

USP is the largest and best characterized family among DUBs, containing approximately 60 members. This family is able to hydrolyze the ubiquitin amide bond through a catalytic triad composed of a cysteine, a histidine, and an asparagine or aspartate residue [35]. The catalytic domain of all USP enzymes shares a common structural arrangement resembling an extended right hand with palm, thumb, and finger subdomains. The catalytic pocket is located between the palm and the thumb, while the fingers could stabilize distal ubiquitin on the protein substrate (Figure 3) [35,36].

### 1.2. USP7 Structure

USP7 represents the best characterized and most studied deubiquitinating enzyme within the USP family. The gene encoding for this 135 kDa (1102 amino acids) cysteine protease is located on chromosome 16p13.2. The USP7 structure can be divided into the following subdomains: (i) an N-terminal polyglutamine (polyQ) stretch region (aa 1–50); (ii) a tumor necrosis receptor-associated factor (TRAF)-like structural domain (aa 53–205); (iii) a central catalytic region (aa 208–560); (iv) five ubiquitin-like domains (UBL1–5) (aa 562–1083); and (v) nineteen amino acid residues at the C-terminus (aa 1084–1102) (Figure 3A) [37,38]. These subdomains are described in detail below:

- The polyQ and TRAF-like domains are highly conserved in humans, mice, and rats and are responsible for the USP7 nuclear localization. Despite the lack of a proper localization signal in this region, it has been postulated that the binding of USP7 to its substrates (commonly nuclear proteins) could favor the translocation into the nucleus [39,40].
- The TRAF-like domain consists of eight β-sheets and one α-helix. This region contains several protein–protein interaction patterns that allow USP7 to recognize the different substrates involved in the ubiquitination pathway, such as MDM2, p53, Epstein–Barr nuclear antigen 1 (EBNA1), viral interferon regulatory factor 4 (IRF-4), latency-associated nuclear antigen (LANA), and testis-specific protein Y-encoded-like 5 (TSPYL5) [41,42].
- The catalytic domain is structurally arranged as an outstretched right hand consisting of thumb, finger, and palm domains. Its structure contains ten α-helices and fourteen β-sheets and includes a conserved catalytic triad composed of Cys223, His464, and Asp481 (Figure 3B) [43]. These three residues are usually in a non-reactive conformation and switch to an active state upon ubiquitin binding. The activation mechanism is facilitated by a region known as the “switching loop,” which brings the catalytic triad residues into proximity, enabling ubiquitin interaction [41].
- The UBL domain consists of five ubiquitin-like ββαβαβ-fold domains possibly involved in substrate recognition and protein–protein interactions [44]. Specifically, UBL 1, 2, and 3 domains interact with infected cellular polypeptide 0 (ICP0), ubiquitin-like with PHD and ring finger domain 1 (UHRF1), and DNA methyltransferase-1 (DNMT1), among others [45]. UBL structural domains 4 and 5 bind to forkhead box O4 (FoxO4) [46]. Notably, the UBL4 domain interacts with the UbcH5c E2 conjugating enzyme, facilitating the transfer of ubiquitin molecules from E1 to the substrate protein, thereby enhancing its stability and mediating various cellular processes [47,48].

UBL domains assume a central role in USP7 regulation, thus affecting several physiological functions such as genome stability, epigenetic regulation, immune response, and viral infection [1]. More specifically, the UBL4–5 tandem, together with the conserved C-terminal peptide, is essential for the allosteric stabilization of the catalytic “switching loop,” thereby enabling full enzymatic activity [43,44]. Additionally, UBL1–3 act as accessory regulatory modules that facilitate cofactor-dependent activation and contribute to substrate recruitment [45]. For these reasons, targeting the UBL domains or altering the stabilization of the inactive conformation via UBL-mediated mechanisms may be feasible strategies for a therapeutic approach [9,17,43].

**Figure 4:**
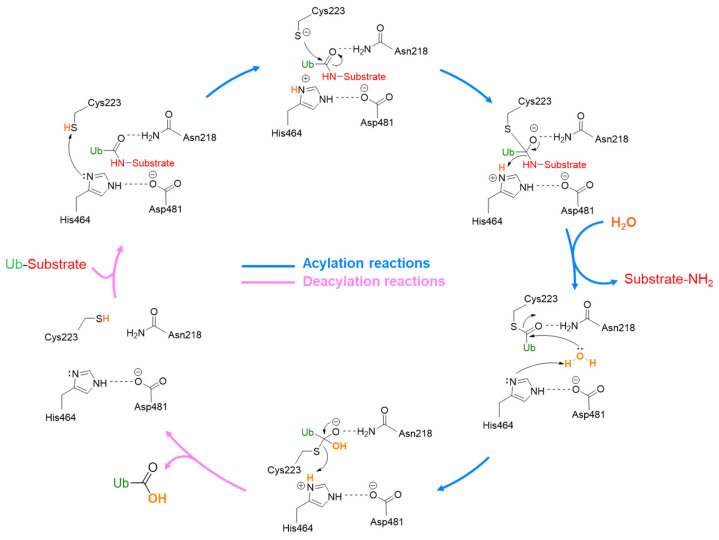
USP7 catalytic mechanism.

The USP7 catalytic mechanism includes different sequential steps (Figure 4). At first, ubiquitin interacts with USP7, and the complex is stabilized by different interactions, including the hydrogen bond between the ubiquitin C-terminal carbonyl and Asn481 amide group. Then, His464 abstracts a proton from the Cys223 thiol group that can attack the isopeptide bond, leading to the substrate release and the thioester intermediate formation. Finally, the thioester group is hydrolyzed by a water molecule and free ubiquitin is released. In this context, Asp481 plays a key role in polarizing His464 and restricting its side-chain rotation [49,50].

Beyond the catalytic triad, various other critical residues within the USP7 catalytic domain influence enzyme specificity and activity. In detail, Ser18 regulates the conformational dynamics of the catalytic cleft, while Phe270 and Phe439 are essential for substrate binding and recognition [51]. Additionally, structural studies revealed that an allosteric site near the catalytic cleft is able to modulate USP7 activity by regulating its conformational changes [52]. Moreover, the hydrolytic activity of the catalytic domain is significantly influenced by the nature of the ubiquitin chains on the substrate protein [30,53].

### 1.3. USP7 Roles and Functions

USP7 has been implicated in the development and progression of various illnesses, including cancer, neurodegenerative disorders, viral infections, and inflammatory disease regression [13,54,55]. Among all the pathologies in which this enzyme is involved, human cancer stands out as the most studied. In this context, USP7 plays a dual role, acting as both a tumor promoter and a tumor suppressor depending on the cancer type [54].

USP7 is critically involved in cancer development, mediating the deubiquitylation (and therefore the stabilization) of oncogenic proteins such as MDM2 and c-Myc, and influencing growth factor TGF-β, which promotes cell survival and proliferation. These actions affect different processes, including cellular DNA damage repair, tumor-related signaling pathway regulation, and immune response, indicating USP7 as a promising target for the development of novel antitumor therapeutics [55,56].

USP7 overexpression has been reported in numerous malignant tumors as a predictive marker of tumor progression and poor prognosis [57]. More specifically, the dysregulation of USP7 expression has been linked to the onset of many malignancies, such as multiple myeloma [58], prostate cancer [16], ovarian cancer [59], cervical cancer [60], glioma [61], colorectal cancer (CRC) [19], liver and brain cancer [9], gastric cancer [2], and head and neck cancer [62]. Additionally, USP7 activity is related to angiogenesis that promotes tumor irroration [15]. Lastly, USP7 is deeply involved in immune system regulation [63]. Specifically, the enzyme can promote cancer immune evasion, enhancing the immunosuppressive functions of regulatory T (Treg) cells and suppressing the activity of effector T (Teff) cells [64]. In recent years, several studies demonstrated a correlation between antitumor drug resistance and USP7 overexpression. Indeed, USP7 pharmacological inhibition could represent a solution for highly aggressive tumors unaffected by drug treatments, indirectly modulating the immune system response [64]. Despite USP7 being mainly involved in cell cycle regulation and tumor progression, other biological pathways have been investigated. For instance, recent studies analyzed the interaction of the enzyme with melanoma-associated antigen D1 (Maged1), a protein specialized in H2A monoubiquitination in the paraventricular thalamus (PVT) that significantly contributes to cocaine adaptive behaviors and transcriptional repression induced by cocaine [65]. For all these reasons, USP7 emerged as a privileged target for the development of new pharmacologically active compounds.

## 2. USP7 Implications in Cancer

As previously discussed, USP7 plays a key role in tumor development and progression, as well as in the regulation of the immune response. In fact, through the deubiquitination process, this enzyme can control the expression of several targets involved in tumor growth and angiogenesis. As anticipated, depending on the substrate and the tumor type, USP7 can act as a tumor suppressor or oncogene [1]. In the next paragraphs, the major USP7 targets involved in cancer development will be discussed.

### 2.1. MDM2 and p53 Regulation

**Figure 5:**
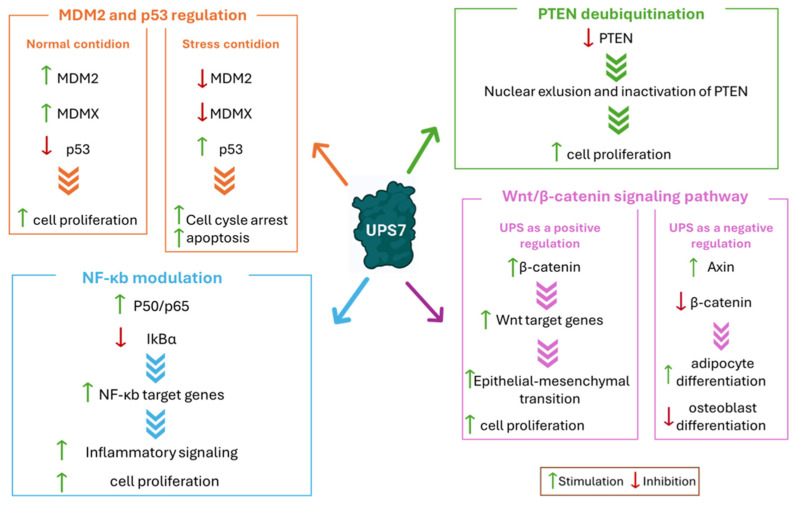
USP7 interaction with the main substrates involved in cancer development. The most relevant pathways affected by USP7 are reported in different colored boxes. Green arrows indicate stimulation; red arrows indicate inhibition.

The tumor suppressor protein p53 is a pivotal factor in the regulation of cell growth, apoptosis, and DNA repair. However, it is highly prone to mutations that shift its function from a tumor suppressor to a tumor promoter due to changes in its spatial conformation [66]. In response to cellular stress, p53 undergoes ubiquitination (beside other post-translational modifications) and proteasomal degradation [67,68]. MDM2 plays a key role in this process, being a substrate of both USP7 and p53 E3 ubiquitin ligase. In normal conditions, MDM2 binds the USP7 TRAF domain even in the presence of an excess of p53 (Figure 5) [69]. Conversely, when the cells are exposed to DNA damage, MDM2 is phosphorylated by the ataxia-telangiectasia mutated kinase, causing its dissociation from USP7. This disconnection triggers MDM2 self-ubiquitination and a subsequent proteasomal degradation (Figure 5). Additionally, USP7 undergoes dephosphorylation by the protein phosphatase Mg^2+^/Mn^2+^-dependent 1G, altering its binding preference to p53. As a result, USP7-mediated p53 deubiquitination leads to cell cycle arrest and apoptosis [10,50]. USP7 helps to stabilize p53 directly by removing its ubiquitin tag or indirectly by deubiquitinating MDM2, a negative regulator of p53. MDM2 binds to the N-terminal deactivation domain of p53, facilitating its ubiquitination and subsequent degradation, thereby inhibiting its transcriptional activity [51]. Furthermore, USP7 interacts with other p53 regulators, such as the Alternate Reading Frame (ARF), which activates p53 [70]. The death domain-associated protein (DAXX) also plays a role in the MDM2/p53 pathway by forming a ternary complex with MDM2 and USP7 [71]. In breast cancer, USP7 cooperates with DAXX in regulating mitosis and taxane resistance, independently from p53 activity [72]. It is well-known that the tumor-suppressing functions of p53, which control cell growth arrest, apoptosis, and senescence, are impaired in about half of human cancers. Furthermore, the amplification and overexpression of p53 negative regulators MDM2 and MDMX are associated with the development of various cancer types [73]. Considering that USP7 is a crucial regulator of the MDM2/MDMX-p53 pathway, its impact on cancer progression and survival has been demonstrated to be linked to the stabilization of MDM2 and MDMX in different tumors such as neuroblastoma (NB) [74], glioma [61], multiple myeloma [58], epithelial ovarian cancer [59], and both lung squamous cell carcinoma and large cell carcinoma [75]. In particular, glioblastoma, one of the most common and aggressive malignant brain tumors, is a good example of the involvement of USP7 and MDM2 in the pathology onset. More specifically, USP7 overexpression leads to the degradation of the retinoblastoma protein, a regulator of the G1 phase of the cell cycle [76]. Furthermore, USP7 can bind to Lysine-specific histone demethylase 1A (LSD1), which further increases the proliferation of the tumor. In fact, when LSD1 is stabilized by USP7, the p53-dependent signaling is suppressed, thus allowing glioma proliferation [77].

### 2.2. PTEN Deubiquitination

The lipoprotein phosphatase PTEN is a tumor suppressor gene frequently mutated in various cancer types. Together with p53, this protein is closely related to tumorigenesis and represents one of the evaluation indexes for tumor prognosis [78]. PTEN’s main function is carried out in the plasma membrane and consists of the inhibition of protein kinase B (Akt), causing the disconnection of the PI3K/Akt/mTOR signaling pathway and leading to cell cycle arrest and consequently to apoptosis [79]. Therefore, PTEN mutation, deletion, or silencing induces tumorigenesis [78,80]. PTEN cytosolic–nuclear translocation mainly occurs through post-translation modification. In detail, monoubiquitination leads to the transport inside the nucleus, while polyubiquitination causes PTEN proteosome-mediated degradation. Finally, USP7-mediated deubiquitination promotes PTEN deactivation through nuclear exclusion (Figure 5). In several malignancies, alterations in USP7 expression cause PTEN nuclear exclusion and lead to disease progression [81]. For example, in prostate cancer, USP7 is often upregulated, promoting PTEN localization in the cytoplasm and not in the nucleus. Additionally, USP7 can affect the stability of the zeste homolog 2 (EZH2) enhancer, a protein involved in the methylation of H3 histone on K27. The inhibition of PTEN nuclear translocation coupled with EZH2 overexpression leads to more aggressive tumors. For these reasons, USP7 inhibition could decrease prostate cancer cell migration, invasion, and proliferation [82]. Similarly, in acute myelogenous leukemia (AML), nucleophosmin 1 (NPM1) regulates PTEN nuclear exclusion by inhibiting its USP7-mediated deubiquitination. Therefore, the PTEN nuclear exclusion induces tumorigenesis and cancer spreading [83].

PTEN deubiquitination plays a pivotal role also in the growth and development of β-cell lymphomas. More specifically, the upregulation of thyroid hormone receptor interactor 13 (TRIP13) induces the intracellular post-translational modification of different proteins including NEK2, MDM2, and PTEN. Several studies reported that higher levels of TRIP13 are associated with poor survival and accelerate tumorigenesis [62]. Differently, in lung neuroendocrine tumors, the loss of nuclear USP7 leads to a reduction in PTEN deubiquitination and to an increase in polyubiquitination, which consequently tags the protein for proteasomal degradation [84].

### 2.3. Wnt/β-Catenin Signaling Pathway

The Wnt/β-catenin cascade is deeply involved in the regulation of embryonic development, apoptosis, tissue homeostasis, cell proliferation, differentiation, migration, and invasion. Thus, dysregulations of this signaling pathway are often associated with the progression of a wide variety of tumors [85]. The most important component of this pathway is β-catenin, a transcription factor which induces the expression of Wnt target genes. Among the different factors that regulate β-catenin levels, ubiquitination proves to be the most relevant one [86]. The complex composed of glycogen synthase kinase 3β (GSK3β), casein kinase 1α (CK1α), adenomatous polyposis coli (APC), and Axin is responsible for β-catenin phosphorylation in the absence of Wnt. The phosphorylated protein is polyubiquitinated by β-transducin repeat-containing protein (βTrCP) E3 ligase and sent to proteasomal degradation [86]. The Wnt/β-catenin cascade is also controlled by deubiquitination. In this pathway, USP7 can either work as a positive or negative regulator (Figure 5). In detail, USP7 acts as a negative regulator of the Wnt/β-catenin axis by deubiquitinating Axin, and therefore inducing the decrease in β-catenin and the suppression of the pathway [87]. Conversely, USP7 can interact with RNF220 E3 ligase to form a RNF220/USP7 β-catenin deubiquitinating complex that exerts positive regulator functions on the Wnt/β-catenin cascade [88]. It was recently demonstrated that CRCs are commonly characterized by a mutation of the β-catenin inhibitory domain (CID) that causes an alteration of the Wnt/β-catenin signaling pathway and consequent β-catenin accumulation [88]. Additionally, in CRCs, USP7-mediated deubiquitination seems to stabilize the DEAD-box helicase 3 X-linked (DDX3X), still promoting β-catenin accumulation. This increased activation of the Wnt/β-catenin axis induces epithelial–mesenchymal transition, a phenomenon strongly related to the advanced stages of the pathology [89]. USP7 pharmacological inhibition suppresses the proliferation of CRC by inducing apoptosis only on cells carrying a mutated CID, suggesting that USP7 acts as a tumor-specific drug target [88].

### 2.4. NF-κb Modulation

The NF-κb transcription factor regulates a series of biological processes involved in the development of multiple pathologies, including autoimmune diseases, inflammation, and hematologic and solid malignancies. NF-κB activates excessive innate immunity and abnormal cell growth, which directly influence tumor development and progression. Additionally, NF-κB also exerts direct anti-inflammatory activity by inhibiting inflammasomes [90]. In order to start gene transcription, NF-κB proteins can dimerize and form (among the others) the p65-p50 heterodimer that was proven to be involved in different pathologies [91]. In non-stimulated cells, the NF-κB dimers bind to NF-κB inhibitors (IκBs) in an inactive state. Upon stimulation, the IκBs subunit β is activated [92], inducing the expression of regulatory genes involved in the regulation of cellular stress, immune response, inflammation, and cell death, differentiation, and proliferation [91,92]. USP7 controls NF-κB through the direct deubiquitination of p65-NF-κB or by indirectly deubiquitinating NF-κB upstream factors (Figure 5) [18]. NF-κB activation seems to be a key mechanism in the bortezomib resistance developed by multiple myeloma. Indeed, recent evidence has demonstrated that USP7 inhibition can stabilize the IκBα inhibitor, causing the arrest of the NF-κB pathway and restoring tumor cell sensitivity to bortezomib. Moreover, USP7 plays a crucial role in multiple myeloma resistance development by binding the NIMA-related kinase 2 (NEK2). This interaction leads to an increase in NEK2 expression, further contributing to NF-κB pathway activation [93]. Additionally, the USP7-NF-κB complex stabilizes the Beclin-1 protein, promoting cellular autophagy and, consequently, contributing to bortezomib resistance [94]. Studies showed that the combination of USP7 inhibitors with bortezomib exhibited a synergistic activity against multiple myeloma, suggesting a promising therapeutic strategy for overcoming resistance to current treatments [95].

### 2.5. Interaction with Insulin Receptor Substrates

Insulin receptor substrates (IRSs) are signaling adapter proteins which mediate the bioactivities of insulin/insulin-like growth factor (IGF) signaling pathways [96]. Tyrosine kinase insulin receptors phosphorylate tyrosine residues of IRSs in response to IGF activation, allowing for the association of IRSs with SH2 domain-containing proteins (e.g., phosphatidylinositol 3-kinase (PI3K) and growth factor receptor-bound protein 2 (Grb2)). These mechanisms cause the activation of other downstream kinases, including Akt and extracellular signal-regulated kinase (ERK), that modulate a wide variety of biological and pathological processes [97]. USP7 is an IRS-associated protein that can protect both IRS-1 and IRS-2 from ubiquitination and consequent proteasomal degradation [15]. Insulin or IGF stimulation activates the PI3K pathway and causes the dissociation of USP7 from IRSs and subsequent IRS degradation [98]. It is well established that IRS-1 and IRS-2 control different functional outcomes in tumor cells. In detail, IRS-1 promotes proliferation through IGF-1R-dependent signaling, while IRS-2 interferes with cell migration, invasion, and glucose metabolism. To prevent excessive signal activation, USP7 inhibits the insulin/IGF signaling. However, the impact of this process on cancer progression is still not clear [99]. Indeed, tumor proliferation is also influenced by other USP7-mediated interactions. For example, recent studies demonstrated that USP7 promoted the spread of prostate cancer through an interaction with the 5-dihydrotestosterone-activated androgen receptor [100].

### 2.6. Cellular Energetics Deregulation

To generate energy for DNA replication and other cellular functions, tumor cells maintain glycolytic activity in both aerobic and anaerobic conditions while reducing the activity of the Krebs cycle and the mitochondrial electron transport system (ETS) [1,9]. Normal cells usually produce thirty-eight ATP molecules through complete aerobic respiration; on the other hand, in cancer cells, normal aerobic respiration is replaced by glycolysis, yielding only two ATP molecules. Hence, to meet their increased energy demand, tumors consume glucose at a significantly higher rate than healthy cells, a phenomenon known as the Warburg effect [101]. Normally, in healthy cells, glucagon triggers the cAMP/PKA signaling pathway, which induces the transcription of gluconeogenic genes. This process activates the expression of different transcription factors, including Forkhead box protein O1 (FOXO1). FOXO1 activity is further enhanced by an unidentified E3 enzyme through monoubiquitination, which promotes the binding of FOXO1 to the promoter regions of the G6Pc and Pck1 genes. Conversely, upon feeding, insulin is released, inhibiting hepatic gluconeogenesis through the PI3K/AKT-mediated phosphorylation and USP7-dependent deubiquitination of monoubiquitinated FOXO1, causing its nuclear exclusion [102].

Another USP7 substrate involved in glucose metabolism is sirtuin 7 (SIRT7), a NAD^+^-dependent class III deacetylase known for its ability to resist glycolytic stress [103]. The SIRT7 enzyme typically binds to the promoter of the G6Pc gene through the ETS-like transcription factor 4 (ELK4). Additionally, SIRT7 can deacetylate H3K1 histone, a key player in the activation of G6Pc transcription, thus starting gluconeogenesis. USP7 regulates SIRT7 by deubiquitinating a lysine residue (namely, K63) on the protein, thereby reducing its activity and suppressing gluconeogenesis [104]. However, despite extensive research, the exact role of USP7 in providing a metabolic advantage to tumor cells remains controversial. Indeed, experimental evidence suggests that the presence of USP7 might be disadvantageous for tumor cell survival. On the other hand, a reduced interaction between USP7 and SIRT7 has been observed in different malignancies, thus promoting gluconeogenesis and proliferation in cancer cells. Therefore, the role of USP7 in tumor cellular energetic deregulation needs further investigation [105].

### 2.7. Other USP7 Implications in Tumors

Breast cancer is commonly divided into three major categories based on hormone receptor expression, namely estrogen receptor (ER) positive, human epidermal growth factor receptor (HER2) positive, and triple-negative breast cancer (TNBC) [106]. In recent years, several studies have shown that the upregulation of Mitogen-inducible gene-6 (MIG-6) induces breast cancer progression with a poor prognosis. More specifically, MIG-6 is able to increase USP7-mediated Hypoxia-inducible factor 1-alpha (HIF-1α) deubiquitination, thus causing its overexpression. This process leads to an enhanced expression of glucose transporter 1 (GLUT1) and, consequently, to a promotion of glucose metabolism and TNBC growth [107]. Furthermore, USP7 can also increase the stability of ATP-binding cassette sub-family B member 1 (ABCB1), thus inducing chemoresistance in TNBC [108].

USP7 also plays a crucial role in the progression of various types of head and neck cancers. In particular, head and neck squamous cell carcinoma (HNSSC) progression was proven to be prompted by the stabilization of the Hippo signaling effector TAZ, induced by the USP7-mediated deubiquitination of its K48-linked polyubiquitin chain [109]. Moreover, in head and neck esophageal carcinoma, the migration and proliferation of tumor cells are mediated by a complex pathway that deeply involves USP7. In detail, Forkhead box protein O6 (FOXO6) upregulation causes an overexpression of USP7, which stabilizes Jumonji domain-containing protein-3 (JMJD3). JMJD3 is able to suppress the transcriptional activity of clusterin by competitively binding to its promoter region and indirectly promotes tumor growth [62].

Finally, in hepatocellular carcinoma (HCC), USP7 seems to increase the expression of the Tripartite Motif Containing 27 (TRIM27) gene by enhancing its stability through deubiqutination. In fact, the activation of the USP7-TRIM27 axis has been associated with an increase in cell proliferation and a poor prognosis [110,111].

## 3. USP7 Role in Angiogenesis and Metastasis

Angiogenesis is regulated by pro- and anti-angiogenic factors (e.g., vascular endothelial growth factor (VEGF) A, thrombospondin-1 (TSP-1)) present in the tumor microenvironment [112,113]. VEGF acts as the key regulator of the angiogenetic mechanism, and its secretion represents a rapid and efficient response to hypoxia in cancer cells. Hypoxia-inducible factors (HIFs) mediate VEGF transcription and consequently activate glycolysis, cell invasion, angiogenesis, and other survival responses [114]. In this context, USP7 plays a pivotal role by deubiquitinating HIFs and other downstream regulators involved in angiogenesis and metastasis processes.

### 3.1. HIF-1α Stabilization

As previously discussed, HIFs mediate angiogenesis by regulating VEGF transcription and can be deubiquitinaned by USP7. In particular, HIF-1α is deeply involved in several signaling pathways that lead to the vascularization of cancer cells [114]. Under normal oxygen conditions, HIF-1α is marked with prolyl hydroxylation at P402 and P564 residues and undergoes proteasomal degradation [115]. Conversely, under hypoxic conditions, USP7 is activated by the HECTH9 protein (polyubiquitination at K443 residue) and consequently stabilizes HIF-1α. The polyubiquitin chain on USP7 represents an anchor point for the CREB-binding protein (CBP), which promotes the acetylation of histone 3 on HIF-1α target genes at lysine 56 [116]. The activity of HIF-1α facilitates the disruption of nuclear export and the degradation of p53, and promotes p53-mediated transcription. As previously mentioned, USP7 binds to both p53 and MDM2 through its TRAF-like domain. The binding site for HIF-1α is also located on the TRAF-like domain, in proximity to the binding sites of p53 and MDM2. Therefore, it has been hypothesized that the HIF-1α/p53/MDM2/USP7 complex can contribute to the regulation of the cellular response to hypoxia [117]. Additionally, HIF-1α is actively implicated in epithelial–mesenchymal transition (EMT) and metastasis [118]. In this context, HIF-1α activity is regulated by FOXO in several ways [119,120,121]. USP7 is one of the antagonistic regulators of FOXO nuclear localization and transcriptional activation. In fact, USP7 plays a pivotal role in the regulation of FOXO4 activity, and for these reasons it has been currently under study as a therapeutic target for diseases associated with dysregulated FOXO4 signaling. However, the precise mechanisms behind the interaction between USP7, FOXO4, and HIF1α is still unclear, and future in-depth studies are needed to discover a specific therapeutic intervention [46,122,123].

### 3.2. EZH2 and Wnt/β-Catenin Pathways Activation

EZH2 is mainly expressed in tumor and vascular cells and is often associated with poor clinical outcomes. This enhancer modulates the expression of specific genes and promotes cell survival, proliferation, and invasion, EMT, and drug resistance in cancers [82]. Furthermore, EZH2 is responsible for the activation of interleukin 6 (IL-6) and tumor necrosis factor (TNF) activation, as well as the interferon gamma receptor 1 (IFNGR1) repression. These events initiate chronic inflammation, which may contribute to tumor development. Additionally, VEGF activation and the subsequent EZH2 high levels stimulate a paracrine circuit that in turn induces angiogenesis [114]. EZH2 is also able to interact with epigenetic modifiers such as LSD1, histone deacetylase 1 (HDAC1), DNA methyltransferase 1 (DNMT1), β-catenin, and the small mother against decapentaplegic (SMAD) proteins SMAD2 and SMAD4, all stabilized by the action of USP7 [124]. The EZH2-USP7 complex forms cytoplasmic chromatin fragments (CCF), which activate the cyclic GMP-AMP synthase-stimulator of interferon genes (cGAS-STING) pathway. This stimulation cascade supports interferon production and further contributes to metastasis formation [125].

As recently demonstrated, USP7 specifically targets EZH2 in prostate and ovarian cancers, thus affecting EZH2-mediated angiogenesis [82]. Therefore, further investigations are necessary to confirm USP7’s involvement in the angiogenetic progression of ovarian and prostate cancer cells and its potential as a therapeutic target [62]. On the contrary, the influence of USP7 on CRC through the activation of the Wnt/β-catenin pathway is well defined [126]. However, despite the large number of Wnt inhibitors identified, only a few drugs have been clinically approved for CRCs; this discrepancy can be ascribed to the inconsistency of the studies on USP7’s effects on the Wnt/β-catenin signaling pathway [127].

### 3.3. NOTCH1 and LSD1 Regulation

The Notch signaling pathway (NOTCH) and the Lysine-specific histone demethylase 1 (LSD1) are two critical ETM and metastasis mediators regulated by USP7 [77,128]. More specifically, USP7 and the histone lysine demethylase JMJD3 stabilize NOTCH1, causing a decrease in H3K27 trimethylation marks, a key point for leukemia initiation and progression. In this context, USP7, NOTCH1, and JMJD3 act in a positive feedback loop where the expression of USP7 is induced by NOTCH1/JMJD3 and turns into the stabilization of oncogenic complexes and target activation [128]. In recent years, several studies have demonstrated that USP7 inhibition can ameliorate the conditions of T cell acute lymphoblastic leukemia patients without toxic effects [129]. Furthermore, LSD1 possesses the ability to mediate gene repression and/or activation by selectively removing the methyl group from mono- or dimethylated histone H3 at lysine 4 (H3K4me1/2) and lysine 9 (H3K9me1/2). In vitro and in vivo studies suggest that USP7 deubiquitination leads to LSD1 overexpression in numerous cancer types (including breast cancer), consequently promoting cancer metastasis [77]. In fact, USP7 deubiquitination is favored by Coactivator Associated Arginine Methyltransferase 1 (CARM1), which stabilizes LSD1. This process causes E-cadherin suppression and the activation of vimentin transcription, which together prompt the invasion and metastasis of glioblastoma and breast cancer cells [130].

## 4. USP7 Influence on Immune Suppression

**Figure 6:**
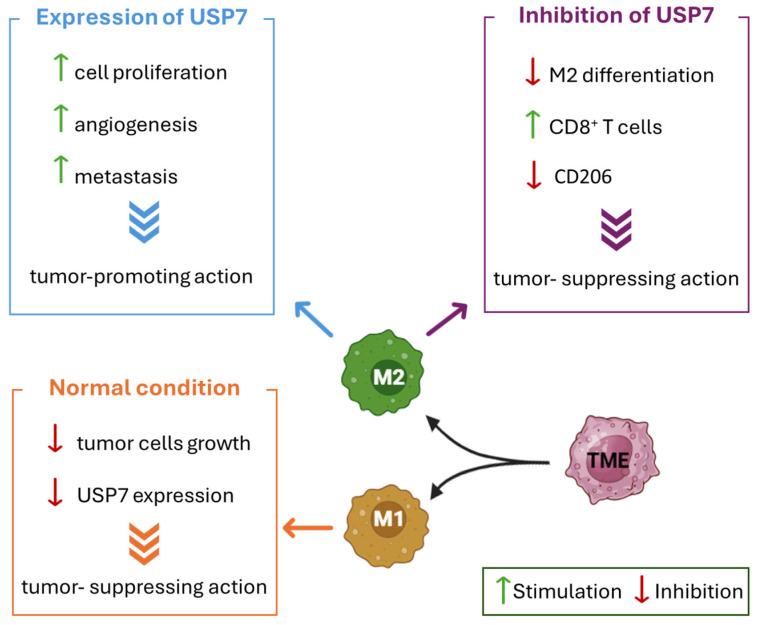
The antitumor immunity suppression in TME mediated by USP7. The effects of USP7 inhibition and activation on M2 macrophages are reported in different colored boxes. The lack of USP7 expression in tumor-suppressing M1 macrophages is summarized in the orange box. Green arrows indicate stimulation. Red arrows indicate inhibition.

USP7 acts as an enhancer of the immunosuppressive functions of Treg cells and, at the same time, as a suppressor of Teff cells, promoting the tumor immune evasion response (Figure 6) [63]. Additionally, USP7 can also interact with specific immune checkpoint inhibitors and interfere with tumor proliferation due to its high expression in tumor-associated macrophages M2 [64,131]. Different pharmacological strategies have been explored to overcome resistance in several types of cancer, especially focusing on Treg inhibition. However, none of them proved to be Treg-specific, and in most of the analyzed cases the risk of inducing toxic inflammation and autoimmunity proved to be too high [132,133,134].

### 4.1. Treg Cells and FOXP3 Activation

Treg cells are the main regulators of immune system responses, being able to control the immunological tolerance to antigens and to maintain peripheral immune homeostasis. The high presence of Treg cells in the tumor microenvironment has been correlated with low survival rates and poor prognosis in several malignancies, including ovarian, breast, gastric, lung, and pancreatic cancers [135]. The FOXP3 transcription factor is the principal regulator of Treg cells, and its mutation causes immune dysregulation in humans [136]. The regulatory mechanism of FOXP3 and its influence on Treg functions have been extensively investigated to establish the role of this transcription factor in tumors’ escape from the immune response [63]. USP7 is critically involved in the regulation of FOXP3 expression [20], as it stabilizes the histone acetyltransferase Tip60 and consequently avoids FOXP3 degradation (Figure 6). Indeed, in Treg cells, Tip60 mediates FOXP3 transcriptional activity through its acetylation and dimerization. In the absence of Tip60, FOXP3 is ubiquitinated and subsequently degraded by the proteasome [137]. By removing polyubiquitin chains from both FOXP3 and Tip60, USP7 enhances FOXP3 activity and protects it from proteasomal degradation [138]. Recent studies suggested that, in FOXP3-expressing Treg cells, the transcriptional activity of the protein is also indirectly regulated by the USP7-p53 pathway [139]. Additionally, Khosravi and collaborators demonstrated that bone marrow-derived mesenchymal stem cells increase USP7 mRNA levels and induce stable FOXP3-expressing Treg cells, which in turn were protected from degradation by the overexpressed USP7 in a positive feedback loop [140]. Therefore, USP7 overexpression enhances the Treg cell-mediated immune suppression and finally leads to tumor growth and proliferation. On the contrary, USP7 inhibition activates antitumor immunity and prompts the effectiveness of conventional immunotherapy strategies [63].

### 4.2. The USP7 Impact on Immune Checkpoint Inhibitors

Immune checkpoint inhibitors are frequently used to improve the antitumor immune response. For example, anti-targeting programmed cell death 1 (PD-1) and programmed cell death ligand 1 (PD-L1) monoclonal antibodies enhance the stimulation of the immune system against cancer cells. Indeed, activated T cells expressed PD-1, which negatively modulates T cell-mediated immune responses [141]. On the contrary, these therapies usually present various issues, such as adverse side effects and resistance development. For these reasons, combination therapies, including the use of USP7 inhibitors, have been widely evaluated in recent years. In fact, the release of anti-inflammatory cytokine IL-10 is one of the common resistance mechanisms underlying anti-PD1 therapy in CRC, and it seems to be suppressed by the USP7 blockage. USP7 inhibition decreases PD-1 and PD-L1 interaction and increases the tumor cells’ sensitivity to the action of T cells, which in turn blocks the tumor growth [142]. Moreover, in gastric cancer, USP7-mediated deubiquitination is responsible for the interaction and stabilization of PD-L1, and the expression of the two proteins seems to be positively correlated. USP7 inhibition sensitized gastric cancer cells to T cell-mediated killing by increasing PD-L1 polyubiquitination and decreasing PD-1/PD-L1 interaction. Additionally, USP7 abrogation leads to tumor suppression through p53 stabilization [142]. In gastric cancer, USP7 is involved in ferroptosis, a programmed cell death mechanism induced by iron-depended lipid-reactive oxygen species. In this context, USP7 stabilizes Heterogeneous Nuclear Ribonucleoprotein A1 (hnRNPA1), which activates the secretion of miT-522 and consequently blocks ferroptosis [143,144]. Overall, these studies highlight the importance of USP7 inhibitors in combination therapies as potential novel therapeutic agents effective against various types of cancer.

### 4.3. USP7 Expression in Tumor-Associated Macrophages and ANXA1

Tumor-associated macrophages are part of the wide variety of cells with a high relevance in inflammation, immunity, and tumorigenesis [131]. These cells can differentiate into M1 and M2 macrophages depending on the different incoming inputs. In detail, M1 macrophages can suppress tumor cell growth, and for this reason they are more present in the early stages of cancer. On the contrary, M2 macrophages are involved in cell proliferation, angiogenesis, and metastasis, and they are abundant when the tumor environment is already established [145]. USP7 is highly expressed in tumor-promoting M2 macrophages, while it is absent in tumor-suppressing M1 macrophages (Figure 6). In recent studies, the effect of USP7 inhibition on M2 expression has been investigated. The arrest of USP7 causes a suppression of M2 macrophage differentiation and activates CD8+ T cells, with a consequent inhibition of Lewis lung carcinoma growth in vivo. Moreover, the block of USP7 reduces the levels of M2-associated marker CD206 and generally suppresses M2 macrophages, without notably cytotoxic effects [131].

Annexin A1 (ANXA1) is another protein-coding gene involved in the suppression of the innate immune system actors [146]; it plays a dual role in adaptive immunity, being able either to activate CD4+ Th cells (and consequently to block the tumor proliferation) or to inhibit their activity and help the cancer cells escape immune suppression [147]. It has been demonstrated that ANXA1 is stabilized by USP7 in HeLa cells; however, the impact of the USP7-ANXA1 axis on antitumor immunity is still unclear and needs further investigations.

To conclude, in recent years, a lot of effort has been spent to clarify the mechanisms through which USP7 modulates the immune system response against tumors. Nevertheless, more research is needed to provide novel cancer treatments which enhance the body’s natural defense [148].

## 5. USP7 Regulation of Genome Stability and Expression

Exposure to endogenous or exogenous agents such as DNA replication stresses, ultraviolet (UV) radiation, reactive oxygen species (ROS), ionizing radiation, and crosslinking agents could result in DNA damage, which represents a common feature in various cancer types. Usually, DNA damage consists of DNA adducts, DNA mismatch, or DNA strand breaks, which are commonly repaired by five major mechanisms: (i) base excision repair (BER); (ii) nucleotide excision repair (NER); (iii) mismatch repair (MMR); (iv) homologous recombination (HR); and (v) non-homologous end joining (NHEJ) [149].

In this context, USP7 preserves the integrity of genomic information by deubiquitinating proteins involved in the five major repair processes and consequently regulating the DNA response and repair system [41]. Moreover, USP7 controls gene expression through epigenetic effects such as DNA methylation or histone modifications that lead to changes in DNA expression patterns [150].

### 5.1. BER and NER

USP7 deubiquitinates and stabilizes specific proteins in the BER and NER excision repair systems, including Mule/ARF-BP1, UVSSA, CSB, and XPC [151]. More specifically, in response to DNA damage, p53 downregulates the Ser18-containing isoform of USP7 (USP7S), decreasing Mule/ARF-BP1 and p53 accumulation to allow for successful BER [51]. On the other hand, USP7 upregulation is necessary to guarantee the two NER mechanisms: transcription-coupled NER (TC-NER) and global genome NER (GG-NER).

Additionally, USP7 plays a pivotal role in UVSSA stabilization and consequently preserves RNA synthesis through successful TC-NER [152]. TC-NER initiation is carried out by the cocaine syndrome proteins CSA and CSB encoded by the cocaine syndrome gene. It has been demonstrated that USP7 inhibition blocks the recovery of CSB under DNA damage induced by UV radiation and, at the same time, reduces RNA synthesis. Conversely, GG-NER started thanks to the XPC-hRad23B/A complex, which can recognize DNA damage. The knockdown of USP7 promotes XPC complex degradation and leads to GG-NER impairment [153].

### 5.2. HR and NHEJ

DNA Double Strand Break (DSB) is the most cytotoxic type of DNA damage since, if not repaired, it may cause chromosomal aberrations [154]. HR and NHEJ pathways can recruit DNA damage mediator proteins, such as the p53-binding protein 1 (53BP1), receptor-associated protein 80 (RAP80), BRCA1, RAD51, and γH2AX, to the damaged chromatin, thus representing the main DNA repair mechanisms in response to DSBs [155,156]. Moreover, DNA damage response is regulated by several proteins that play a pivotal role in the repair processes by promoting the accumulation of BRCA1 and 53BP1, the major repair factors in the HR and NHEJ pathways [157]. In this context, USP7 acts as a crucial mediator of the HR and NHEJ repair mechanisms by directly regulating the stability of DNA repair proteins or by indirectly affecting their activities through deubiquitination [158]. In detail, USP7 down-expression leads to a decreased expression of RNF168, which ultimately arrests the accumulation of BRCA1 and 53BP1 [159].

Additionally, USP7 influences the activity of RNF169 E3-ligase and displaces the binding of 53BP1 and RAP80 at the DNA damage site. The entrance of the cells into senescence and apoptosis is bypassed when RNF169 accumulation occurred due to the modification of RAD-51-dependent homologous recombination [160].

Two other crucial players able to initiate and amplify the DNA damage response are the MRE11-RAD50-NBS1 (MRN) complex and the mediator of DNA damage checkpoint protein 1 (MDC1). In particular, MDC1 contributes to the accumulation at DNA lesions of 53BP1 and BRCA1 and induces the ubiquitination of γH2AX and H2A by recruiting RNF8 and RFN168 [161]. The interaction between the MRN complex and USP7 deubiquitinates and stabilizes MDC1. On the contrary, USP7 inhibition leads to the suppression of both HR and NHEJ repair pathways caused by the reduction in the MDC1-mediated accumulation of BRCA1 [162]. Increased USP7 levels seem to enhance the survival rate of cervical cancer cells through MDC1 stabilization and consequently improve resistance to DNA damage [163].

### 5.3. Cell Cycle Arrest

Cell cycle arrest and apoptosis represent other more drastic DNA damage response strategies. As previously discussed, USP7 plays a crucial role in cell cycle regulation, particularly through its interaction with p53 and MDM2. However, USP7 can also interact with several other substrates involved in this process. Despite its role appearing controversial in relation to its oncogenic activities, the enzyme can regulate cell cycle checkpoint proteins by altering their stability and therefore promoting cell cycle arrest [164].

The Ataxia Telangiectasia and Rad3-related protein Checkpoint kinase-1 (ATR-Chk1) pathway is one of the signal cascades involved in cell cycle arrest. More specifically, the phosphorylation of Chk1 (at serine residues 317 and 345) causes its dissociation from chromatin and the subsequent phosphorylation of other targets, such as Cell Division Cycle 25A (CDC25A). The process concludes with cell cycle arrest at the S and G2 phases [165]. USP7 stabilizes Chk1, claspin, and CDC25A, which distinctly affect CDK-dependent cell cycle progression. Indeed, the stabilization of CDC25A, and consequently its accumulation, may be exploited by cancer cells to continue cell cycle progression even in the presence of DNA damage. Additionally, by stabilizing CDC25A, USP7 ensures a constant protein level, which provides the cell the ability to resume its cell cycle after DNA repair [69,166].

Among the USP7 substrates involved in the cell cycle processes, Coiled-coil domain-containing protein 6 (CCDC6) proved to carry out an important role in non-small cell lung cancer (NSCLC). In particular, the overexpression of CCDC6 increases during the G2 phase and decreases during mitosis. Since USP7 is able to enhance CCDC6 stability, silencing the enzyme leads to the turnover of CCDC6 and cell cycle arrest [167]. In NSCLC tumors, USP7 can also deubiquitinate the TRAF-like domain of serine/threonine kinase Raf-1. By reducing Raf-1 activity, USP7 blocks the ERK signaling pathway, thereby regulating the tumor proliferation of NSCLC [84].

Moreover, USP7 appears to play a key role in lung cancers resistant to Paclitaxel through the regulation of two proteins involved in cell mitosis: Serine/threonine-protein kinase or polo-like kinase 1 (PLK1) and BUB3 Mitotic Checkpoint Protein (BUB3). In fact, recent studies have shown that a combined treatment with a PLK1 inhibitor (Volasertib) and a USP7 inhibitor (**P22077**, reported below) induces the death of Paclitaxel-resistant cancer cells [168].

## 6. USP7 Inhibitors

As reported above, several recent studies highlighted the attractiveness of USP7 as a valuable and innovative target for anticancer therapy and prompted extensive research efforts towards the development of potent and selective USP7 inhibitors. Up to now, nearly 160 small molecules able to pharmacologically inhibit USP7 have been discovered [54,169].

However, despite several USP7 inhibitors having been reported in the scientific literature, the search for potent and selective compounds is still challenging and, to date, no molecules have been approved as commercial drugs. Indeed, many molecules are not selective for USP7 and may also react with other DUB enzymes, making the substrate-specific identification difficult. In addition, most of the molecules exhibit a low inhibition potency, with only a few inhibitors presenting an IC_50_ value in the nanomolar range [15,170,171,172]. Another limiting factor is the difficulty in carrying out cellular assays due to unfavorable pharmacokinetic properties and the poor solubility of the molecules. For these reasons, only a small number of USP7 inhibitors have been tested in vivo [15].

**Figure 7:**
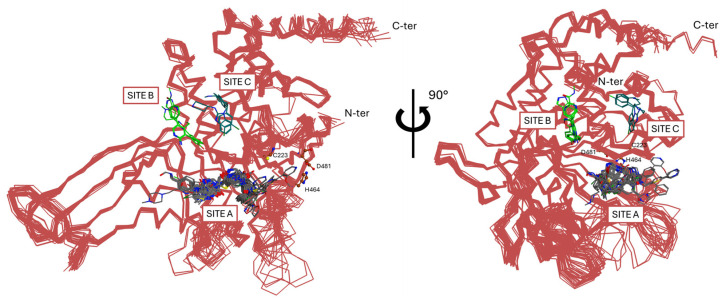
Superimposition of the reported crystal structures of USP7 bind to allosteric inhibitors (Site A and Site B) and allosteric activators (Site C).

Based on their interaction with the enzyme, three main categories of USP7 inhibitors can be distinguished, namely covalent inhibitors, noncovalent allosteric inhibitors, and covalent allosteric inhibitors [169]. According to the crystallographic data available so far, most allosteric inhibitors would interact with the enzyme in a region adjacent to the catalytic triad between the thumb and the palm (Site A, Figure 7). Additionally, two binding zones have been detected in the thumb subdomain above Site A and the catalytic triad; more specifically, Site B (Figure 7) hosts a couple of allosteric inhibitors, while Site C (Figure 7) is involved in the binding of allosteric activators (vide infra). Interestingly, in addition to the previous categories, a group of natural inhibitors extracted from sea sponges have been discovered [173,174,175].

### 6.1. Covalent Inhibitors

**Figure 8:**
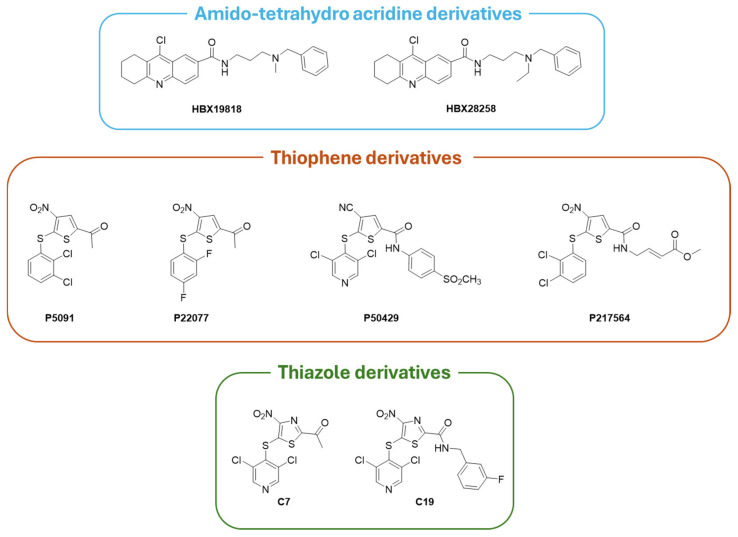
Chemical structures of selected USP7 covalent inhibitors.

Covalent inhibitors interact with the USP7 catalytic domain through the formation of a covalent bond with the catalytic Cys223 residue. This type of binding prevents the conformational transition of the enzyme, blocking its deubiquitinase activity irreversibly [169]. The amido-tetrahydro acridine derivatives **HBX19818** and **HBX28258** (Figure 8) act as USP7 covalent inhibitors, resulting in **HBX19818** being effective against both CRC and chronic lymphoid leukemia (CLL), whereas **HBX28258** is active only against CRC [15].

Tri-substituted thiophenes **P5091**, **P22077**, **P50429,** and **P217564** (Figure 8) irreversibly block USP7 activity [58,171,176]. In detail, **P5091** showed antitumor activity in vivo against multiple myeloma (MM.1S xenograft model), CRC, glioblastoma, and esophageal squamous cell carcinoma thanks to the activation of the p38 MAPK signaling pathway [74,177]. Conversely, **P22077** and **P50429** inhibitors act by modifying Cys223 and altering the orientation of the surrounding loops, thus preventing the alignment of the substrate towards the catalytic site; moreover, **P22077** and **P50429** are active on NB and CRC, respectively [178,179]. **P217564** is a potent second-generation USP7 covalent inhibitor with enhanced suppressive action on Treg cells and, consequently, anticancer activity [180]. Based on the already known thiophene scaffold inhibitors, additional molecules active on USP7 have been developed as thiazole derivatives (i.e., derivatives **C7** and **C19**, with IC_50_ of 0.67 and 1.35 µM, respectively, Figure 8). These USP7 thiazole inhibitors turned out to be more potent than their thiophene congeners, and both compounds showed a significant activity against CRC [178].

### 6.2. Noncovalent Allosteric Inhibitors

**Figure 9:**
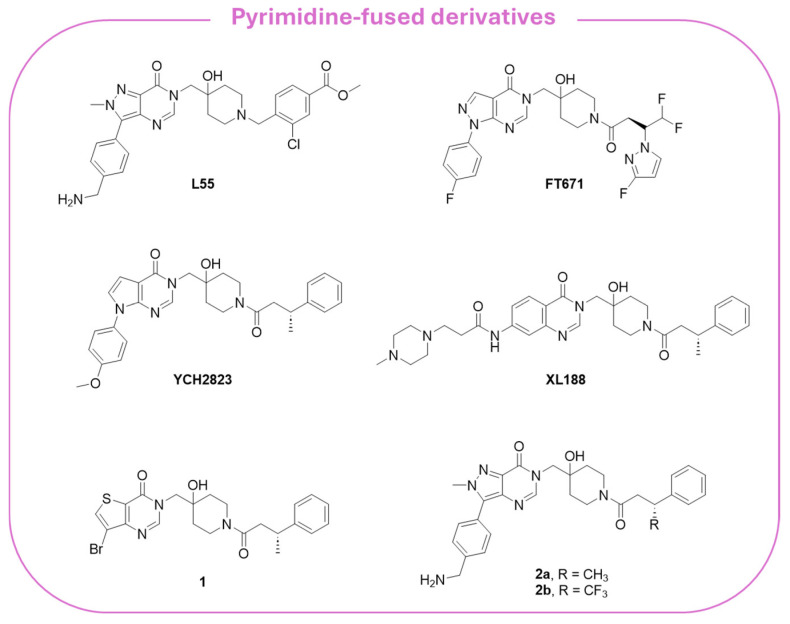
Chemical structure of the pyrimidine-fused USP7 noncovalent allosteric inhibitors.

Reversible allosteric noncovalent inhibitors bind regions adjacent to the catalytic triad and prevent the proper alignment of the three catalytic amino acids, blocking ubiquitin binding [169]. Among the different noncovalent allosteric inhibitors, pyrimidine-based compounds are the most studied. In particular, derivatives **L55** and **FT671** (Figure 9) are characterized by a pyrazole–pyrimidine structure, **FT671** being a structural optimization of **L55** [180,181]. The USP7-specific **FT671** inhibitor (IC_50_ = 52 nM) is active against CRC and breast cancer, blocking the proliferation of MM.1S cells with an IC_50_ value of 0.33 µM, as determined by the CellTiter-Glo assay. The crystal structure of the USP7 catalytic domain complexed with **FT671** (PDB code: 5NGE) showed the ability of the inhibitor to alter Phe409 orientation without affecting the positions of Tyr465 and Tyr514 [181].

Taking **FT671** as a lead compound, Cheng and collaborators synthesized a series of novel pyrrolo-pyrimidine derivatives and identified **YCH2823** (Figure 9) as a potent USP7 inhibitor (IC_50_ = 0.50 µM) with a remarkable anti-proliferative impact on various tumor cells [182]. At the cellular level, **YCH2823** modulates the turnover of multiple substrates, leading to p53 stabilization, p21 induction, and a decreased expression of Rad18 and DNMT1. These interactions collectively result in cell cycle arrest and apoptosis. Importantly, **YCH2823** displays strong antitumor activity across a broad spectrum of cancer cell lines, including those harboring both wild-type and mutated TP53. Moreover, mTOR inhibitors enhanced **YCH2823** efficacy, highlighting a promising therapeutic strategy to extend the clinical utility of USP7 inhibitors, particularly in MYCN-amplified cancers [182].

The quinazolin-4-one analog **XL188** (Figure 9) is structurally related to the pyrazole–pyrimidine **FT671**, and the two inhibitors share amide groups on the piperidinol scaffold. More specifically, **XL-188** emerged from a structure-guided optimization campaign that converged on a rigid quinazoline/heteroaromatic core [183]. A conserved multi-heteroatom motif on the core forms key hydrogen bonds with backbone and side-chain polar residues in the pocket; this hydrogen-bond network proved to be critical for potency. Additionally, a rigid core scaffold allowed the correct orientation of interacting groups, showing that flexible analogs reduced USP7 inhibition.

**Figure 10:**
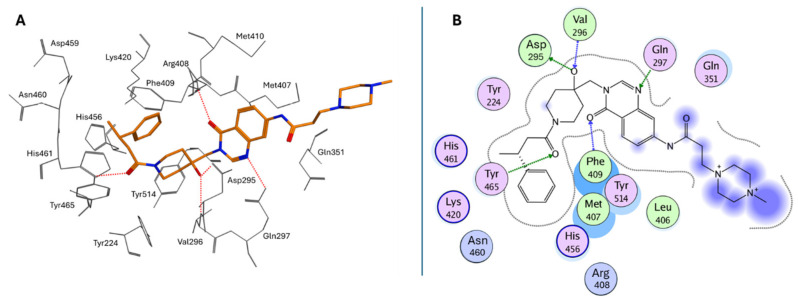
(**A**) Bioactive conformation of **XL188** in USP7 binding site; (**B**) Ligplot for the USP7/**XL188** X-ray complex.

Peripheral substituents projecting into adjacent solvent channels were tuned to improve cellular potency and ADME properties without perturbing core contacts. As a result, modest increases in the polar surface area or polar solubilizing groups enhanced cell activity, whereas an excessive lipophilicity degraded free-drug exposure [183]. As emerged from the X-ray structure of **XL188** complexed with the USP7 catalytic domain (PDB: 5VS6; Figure 10A), the inhibitor occupies the S4 and S5 subsites, about 5.5 Å away from the catalytic triad. The compound assumes an extended conformation, being the hydroxypiperidinyl-quinazolinone portion involved in multiple hydrogen bonds with Asp295 (side-chain carboxylate), Val296 (backbone NH), Gln297 (side-chain amide), Phe409 (backbone NH), and Tyr465 (OH side chain) (Figure 10B). The phenyl ring of **XL188** is buried in the S4 pocket and is in contact with the aromatic rings of Tyr514, His456, and Phe409, and the aliphatic chains of Lys420 and Arg408. Notably, the side chain of Phe409 flips to reveal the hydrophobic pocket, a conformational rearrangement also observed upon binding of ubiquitin [43]. Interestingly, the **XL188** inhibitor partially protrudes into the channel normally occupied by the ubiquitin C-terminal tail and protects the α-4/5 regions surrounding the S4-S5 pocket. This peculiar conformation made **XL188** unable to bind other USP isoforms, therefore resulting in it being highly selective against USP7, with an inhibitory activity in the nanomolar range (IC_50_ = 90 nM) [184]. Furthermore, the proximity of **XL-188** to the catalytic cysteine suggested a tractable vector for the design of covalent inhibitors. Thus, the decoration of the central core with appropriately positioned electrophile groups can convert a noncovalent lead into an irreversible analog with an improved potency [183,184].

Through fragment-based methods, Gavory and coworkers identified thieno-pyrimidine **1** and pyrazolo-pyrimidines **2a,b** (Figure 9) as selective USP7 allosteric inhibitors. The derivatives exhibited IC_50_ values in the sub-micromolar range and proved to significantly inhibit cell growth in p53-mutant cancer cells, being more active than several PROTAC congeners derived thereof [185].

**Figure 11:**
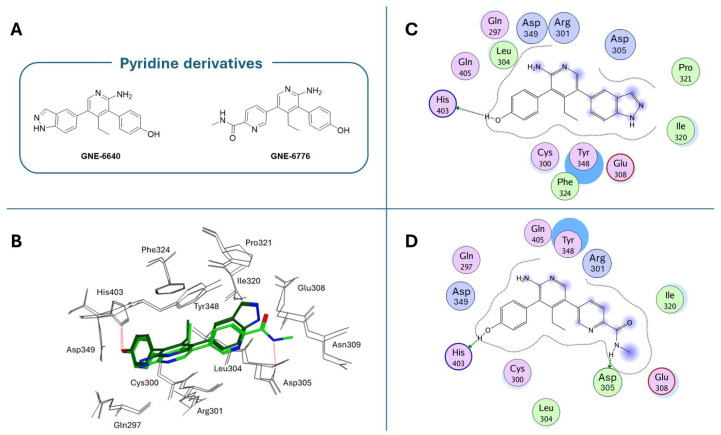
(**A**) Chemical structures of pyridine-based USP7 noncovalent allosteric inhibitors; (**B**) binding poses of **GNE-6640** (dark green) and **GNE-6776** (light green) in USP7 binding site; (**C**) Ligplot for the USP7/**GNE-6640** complex; (**D**) Ligplot for the USP7/**GNE-6776** complex.

**GNE-6640** and **GNE-6776** compounds (Figure 11A) represent an alternative class of USP7 allosteric inhibitors characterized by a highly substituted pyridine scaffold. The GNE series emerged from an NMR-fragment campaign and iterative structure-guided optimization that converged on a small, rigid heteroaromatic core that docks into an acidic pocket of the USP7 catalytic domain. Chemical elaboration around this core showed that a scaffold presenting an H-bond donor/acceptor triad was necessary for the potent attenuation of ubiquitin binding, whereas flexible linkers or over-polarization reduced biochemical potency and cell activity [171]. Peripheral substituents that projected into nearby solvent channels improved the cellular potency and ADME properties (solubility, plasma exposure) without perturbing the core pocket interactions; conversely, an excessive lipophilicity increased protein binding and worsened free-drug exposure in vivo. **GNE-6776** embodies PK-balanced modifications that afforded superior cellular efficacy, oral bioavailability, and xenograft activity relative to earlier analogs (including **GNE-6640**), indicating that modest changes to terminal substituents and the polar surface area were fundamental for in vivo translation. These observations support a lead-optimization strategy that preserves the core H-bonding motif for the acidic pocket, rigidifies the scaffold to minimize entropy loss, and fine-tunes peripheral substituents to balance potency with favorable free-drug exposure for in vivo efficacy [171]. As emerged from crystallographic studies (PDB codes: 5UQV and 5UQX), these molecules bind USP7 in a pocket located at the interface of the palm, finger, and thumb subdomains, approximately 12 Å away from the catalytic triad (Site B; Figure 7). This binding site is not conserved in other classes of DUBs, and it was exploited to improve the selectivity against the USP7 isoform, where it seemed to play a role in the ubiquitin binding [183]. In both crystallographic complexes, the inhibitors assume an extended conformation (Figure 11B), with the phenol OH group hydrogen bonded to the His403 side chain (Figure 11C,D). The substituents at the 5-position of the aminopyridine scaffold (i.e., indazole for **GNE-6640** and pyrimidine carboxamide for **GNE-6776**) point toward the solvent, protruding between α5 and α6 helices. Furthermore, the USP7-**GNE-6640** complex is further stabilized by van der Waals interactions between the indazole and the amino acid residues on α5 and α6, while the NH atom of the **GNE-6776** carboxamide-moiety is in contact with Asp305. Finally, the co-crystal structures of both molecules revealed their ability to sterically inhibit ubiquitin binding and prevent the transition of the USP7 catalytic domain α5 helix to the active conformation [171]. **GNE-6640** shows an IC_50_ value of 0.75 µM on USP7, causing apoptosis and being more active than **GNE-6776** (IC_50_ = 1.35 µM), which triggers cell cycle arrest [171].

**Figure 12:**
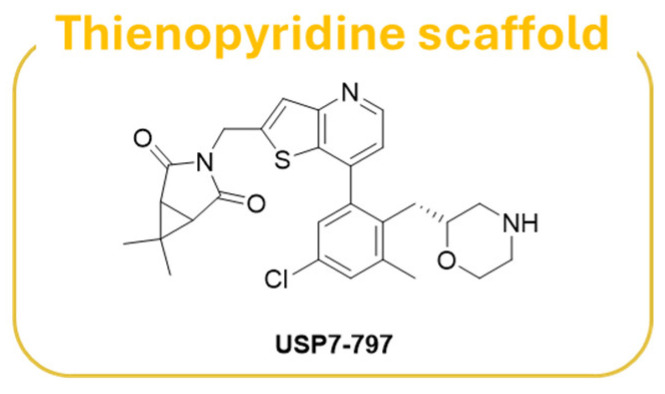
Chemical structure of thienopyrimidne derivative **USP7-797**.

Thienopyridine **USP7-797** (Figure 12) has been identified as a USP7 allosteric inhibitor endowed with an optimal balance between cellular potency and pharmacokinetic properties in vitro [186]. The inhibitor was able to reduce the viability of multiple TP53 wild-type cell lines, including several hematologic cancers, and MYCN amplified NB cell lines, as well as a subset of TP53 mutant cell lines [186].

### 6.3. Covalent Allosteric Inhibitors

**Figure 13:**
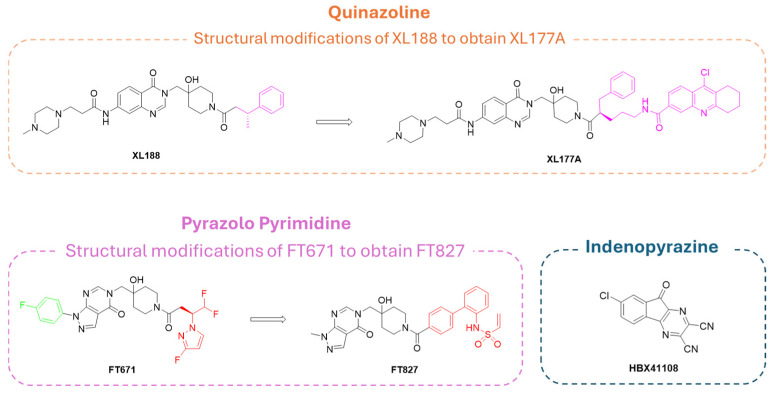
Chemical structures of reported USP7 covalent allosteric inhibitors.

Covalent allosteric inhibitors bear reactive functionalities able to form covalent interactions with the target at a different site from the catalytic triad. In comparison with the other class of USP7 inhibitors, these compounds are poorly attractive due to the lack of selectivity, leading to off-target effects. For instance, the cyano-indenopyrazine **HBX41108** inhibitor (Figure 13) can bind the enzyme–substrate complex in a pocket near the ubiquitin binding site [187]. However, **HBX41108** seems to lack selectivity (e.g., IC_50_ (USP7) = 0.42 µM; IC_50_ (USP8) = 0.96 µM), as its 2,3-dicyano-pyrazine substructure is highly reactive and can covalently modify all DUBs at a 5 µM concentration [138,187,188,189].

Differently, quinoline derivative **XL177A** (Figure 13), synthesized from its congener **XL188**, was more effective on USP7, being able to form both noncovalent allosteric and covalent orthosteric interactions. In fact, **XL177A** can covalently bind Cys223 residue, enhancing the exchange in the α2 to α4 region of USP7 [34].

Finally, pyrazolo-pyrimidine **FT827** (Figure 13), prepared from the **FT671** precursor, can play a dual role, acting as both a noncovalent and covalent allosteric inhibitor. However, the lack of specificity for the different USP isoforms reduces the pharmaceutical attractiveness of this compound [181]. In fact, despite the presence of the pyrazolo-pyrimidine core, designed to specifically target USP7 S4 and S5 sub-pockets, the pyrazole nitrogen substituents seemed to be highly interactive with different amino acids in the catalytic pocket of all USP isoforms, leading to a lack of selectivity [33].

### 6.4. USP7 PROTACs

Targeted protein degradation (TPD) is an emerging therapeutic approach with the potential to tackle disease-causing proteins that are proven to be highly challenging to target with conventional small molecules. Among TPD strategies, proteolysis-targeting chimera (PROTAC) degraders represent the most consolidated approach [190]. PROTAC heterobifunctional small molecules consist of two ligands joined by a linker; one ligand binds to a protein of interest, while the other warhead recruits an E3 ubiquitin ligase. Simultaneous binding of the protein of interest and ligase by the PROTAC induces the ubiquitylation and subsequent degradation of the protein through the UPS; after the proteolysis of the protein of interest, PROTAC is released and can mediate the degradation of another complex [191,192].

**Figure 14:**
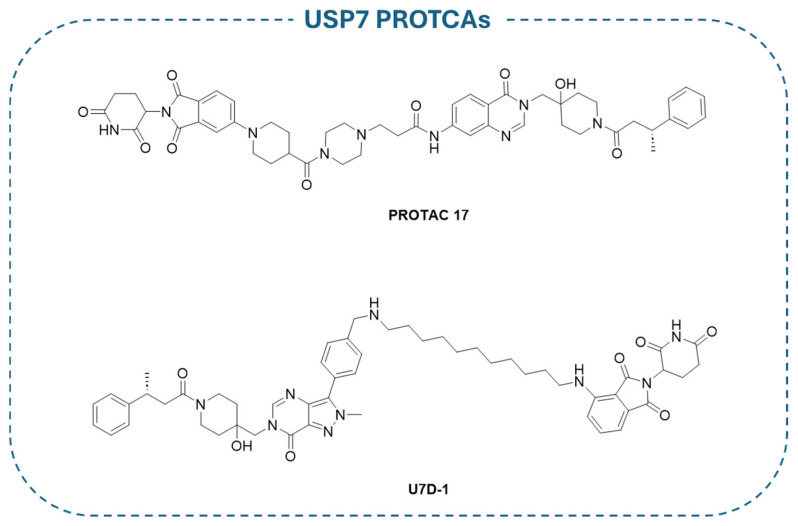
Chemical structure of reported USP7 PROTACs.

Based on XL188 noncovalent allosteric inhibitor, **PROTAC 17** (Figure 14) has been developed. This compound is one of the most promising PROTACs for USP7 (IC_50_ = 1.6 ± 0.3 µM), consistently showing an activity in USP7-dependent cancer cells [193]. Similarly, **U7D1** (Figure 14) has been designed from the potent non-competitive USP7 inhibitor **2a** (Figure 9) and demonstrated relevant inhibitory activity (IC_50_ = 6 nM) against cancer cells bearing p53 mutations non-sensitive to other USP7 inhibitors [194]. Both **PROTAC 17** and **U7D1** were designed by linking well-known selective USP7 inhibitors to a Cereblon (CRBN) E3 ligase ligand through different decorated linkers. As demonstrated by proteomic studies, the nature of the CRBN ligand and the length of the linker directly influence USP7 degradation. Significantly, the PROTAC derivatives were more effective against cancer cells in comparison with the treatment of the sole USP7 inhibitor [193,194].

### 6.5. Natural Inhibitors

**Figure 15:**
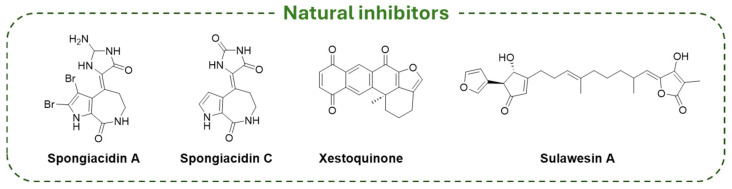
Chemical structure of the USP7 natural inhibitors.

Despite the majority of USP7-targeting derivatives being synthetic compounds, there are a few examples of natural inhibitors. In detail, pyrrole alkaloids isolated from marine sponges (i.e., **Spongiacidin A**, **Spongiacidin C**, **Xestoquinone**, **Sulawesin A**; Figure 15) showed USP7 inhibitory activity. Thus, **Spongiacidin A** was the first natural molecule extracted from a natural source with micromolar USP7 inhibitor activity (IC_50_ = 8.5 µM) [195]; then, **Spongiacidin C** was isolated from the marine sponge *Stylissa massa* and showed an improved potency against the enzyme (IC_50_ = 3.8 µM) [173]. The pentacyclic quinone **Xestoquinone**, isolated from the marine sponge *Petrosia alfiani*, showed multiple biological activities, including a significant inhibitory activity on USP7 (IC_50_ = 0.13 µM) [174]. Finally, **Sulawesin A** is a mixture of four diastereomers (namely, 5R,9R,18R, 5R,9R,18S, 5S,9S,18R, and 5S,9S,18S) isolated from a marine sponge, *Psammocinia* sp. The four isomers are furano-sesterterpene tetronic acids and are USP7 inhibitors with an IC_50_ value of 2.8 µM [175,196].

## 7. USP7 Activators

As previously discussed, USP7 can play several roles in cancer progression and metastasis, acting either as an oncogenic protein or tumor suppressor based on the targets involved and the cancer type. Although a wide variety of USP7 inhibitors have been developed over the last decade, USP7 activators may also represent useful molecules for anticancer therapy [44,52]. It has been demonstrated that the USP7 C-terminal portion can interact with the activation cleft in the catalytic domain, stabilizing the active conformation of the enzyme and thereby enhancing the enzyme’s deubiquitinase activity [43].

**Figure 16:**
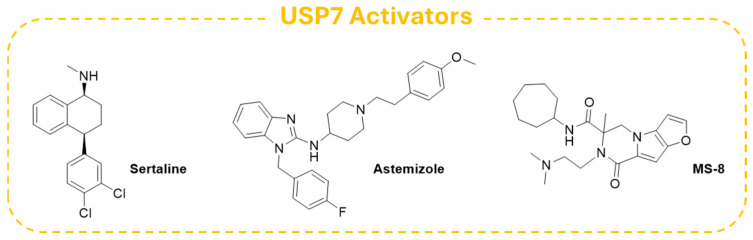
Chemical structures of USP7 allosteric activators.

In this context, Shi and colleagues carried out a virtual screening on a panel of FDA-approved drugs and identified the antidepressant **Sertraline** (Figure 16) and the antihistamine drug **Astemizole** (Figure 16) as selective USP7 agonists. Further biochemical and biophysical experiments confirmed that the two derivatives could bind the switching loop region of the USP7 C-terminal domain, displaying an agonistic potency in the low micromolar range. Additionally, both **Sertraline** and **Astemizole** proved to enhance the deubiquitinase activity of selected USP7 pathogenic mutants, and the antihistamine compound was also able to upregulate the enzyme in mutants associated with Hao–Fountain Syndrome (HAFOUS) in a cell-based assay. Despite the side effects associated with the intrinsic action of these drugs, the obtained results highlighted the importance of the development of novel USP7 agonists for the treatment of diseases associated with the loss of function of the enzyme [197].

**Figure 17:**
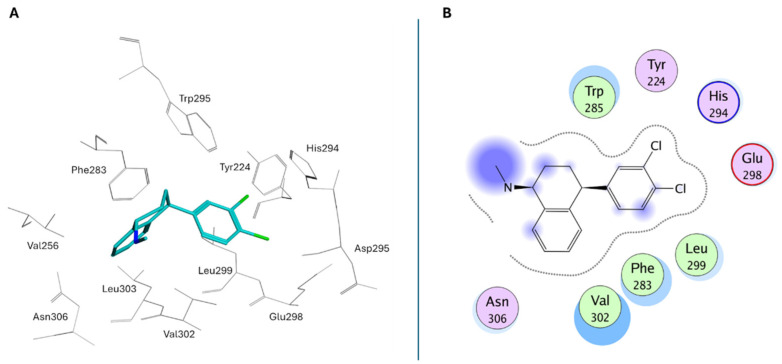
(**A**) Binding pose of **Sertraline** in USP7 activation cleft; (**B**) Ligplot for the USP7/**Sertraline** crystallographic complex.

The crystal structure of the USP7/**Sertraline** complex revealed that the compound binds in a cleft composed by the switching loop (Phe283–Asp295) and α-helix α5 (Val296—Met311) (site C, Figure 7). **Sertraline** assumes a protruded bioactive conformation (Figure 17A, PDB: 9IJU) and interacts with hydrophobic residues such as Phe283, Trp285, His294, Leu299, and Val302 through van der Waals contacts, while the methylamino substituent is solvent exposed (Figure 17B). Additionally, the two aromatic structures of the derivative would occupy the S1 and S2 sub-pocket in the activation cleft, consequently stabilizing the active conformation of the enzyme and enhancing its deubiquitinase activity [197].

With the aim of discovering novel small molecules for the treatment of HAFOUS, Maisonet and coworkers carried out a large-scale screening and identified the compound **MS-8** (Figure 16) as a potential USP7 activator. The molecule can mimic the USP7 mechanism of allosteric autoactivation by targeting the enzyme in the allosteric C-terminal binding pocket. Furthermore, **MS-8** induced an increase in intracellular levels of the USP7 downstream substrates (e.g., TRIM27 and p21) in wildtype HCT116 cells without exhibiting cytotoxic effects. This evidence supported the use of small molecule activators for the treatment of HAFOUS and other related diseases. Interestingly, **MS-8** is currently under study for the development of new DUB Targeting Chimeras (DUBTACs), heterobifunctional derivatives able to stabilize the levels of the protein of interest, preventing its proteasomal degradation [198].

The discovery of **MS-8,** together with the interesting properties shown by the clinically used molecules **Sertraline** and **Astemizole,** laid the groundwork for the development of novel anticancer therapies that will substitute the predominant inhibitory approach [199]. Additionally, cheminformatic and computational studies have discovered innovative druggable allosteric pockets that influence the dynamic switching loops and consequently increase the USP7 activity [200]. Overall, this preliminary evidence establishes a proof of concept for USP7 potentiation benefits, but medicinal chemistry optimization as well as more in vitro validation are still needed before pre-clinical application [198].

## 8. Conclusions

DUBs are fundamental regulators of the ubiquitin–proteasome system, playing a key role in maintaining protein homeostasis. Among the various families of DUBs, USPs represent the largest and the most studied group, with USP7 (also known as HAUSP) emerging as a crucial player in multiple cellular processes. Indeed, USP7 modulates a wide array of substrates by removing ubiquitin moieties, thereby influencing their stability, activity, and subcellular localization. Its regulatory functions span critical biological pathways, including the DNA damage response, immune signaling, epigenetic modifications, cell cycle progression, and apoptosis.

**Figure 18:**
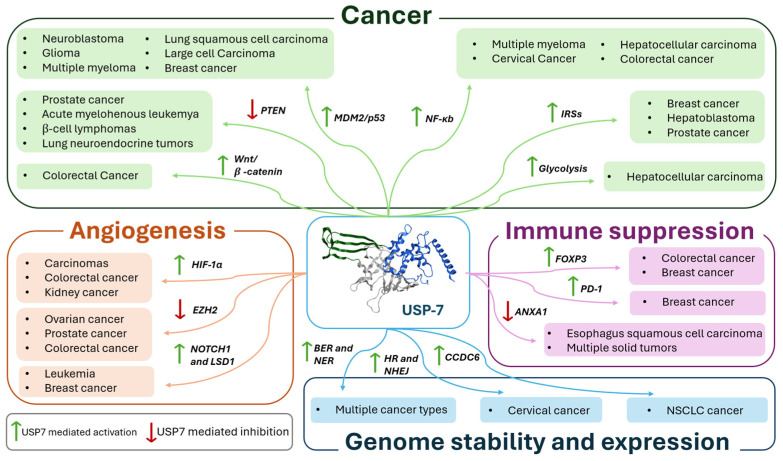
Schematic representation of the pathways and related pathologies in which USP7 is involved.

Interestingly, USP7 exerts its action by stabilizing both oncogenic and tumor suppressor proteins, such as MDM2, p53, PTEN, FOXP3, β-catenin, and NF-κB (Figure 18). This dual activity reflects its complex role in cancer biology, where USP7 can either promote or suppress tumor development depending on the cellular context. Aberrant expressions or activity of USP7 have been implicated in a broad range of pathological conditions, including cancer, neurodegeneration, viral infections, and autoimmune disorders, consequently positioning it as an attractive pharmacological target.

As summarized in Table 1, numerous small-molecule USP7 inhibitors have been reported in recent years, targeting both catalytic and allosteric sites inside the catalytic region of the enzyme. Additionally, few allosteric activators have been synthesized, showing interesting biological results. Collectively, the reported compounds have demonstrated encouraging in vitro results, with some of them showing a high selectivity and potency. However, despite the growing interest and promising outcomes of in vitro and in vivo studies, no USP7 inhibitors have reached clinical approval yet. In fact, the dual activity of the enzyme and its ability to act on multiple targets within the same tumor make the pharmacological intervention very difficult, often leading to off-target or unwanted side effects [201].

Additionally, the role of UBL domains in the development of USP7-related diseases is still unclear. These domains are challenging to target with small molecules due to their shallow binding surfaces and dynamic conformational flexibility, which complicate drug design. Understanding UBL-mediated interactions remains crucial for developing effective therapeutics that modulate USP7 activity [109,202].

Finally, despite, on the one hand, the discovery of selective USP7 activators potentially being a game changer for the therapeutic approach to the enzyme, on the other hand, the collected results are still too weak to reach clinical application, and more SARs studies, as well as computational analysis, are needed to better understand the mechanisms of action of this class of compounds [199,200].

For these reasons, future research efforts should continue to focus on improving our understanding of USP7 substrate specificity and context-dependent roles in disease. In fact, a deeper comprehension of USP7 molecular mechanisms will be essential to guide the rational design of more effective and selective inhibitors.

The data reported here were collected from different databases (Scifinder, Web of Science, Scopus, Google Scholar, and Pubmed) using “Deubiquitinases” or “USP7” and “inhibitors” as keywords, and considering publications (i.e., patents, reviews, research articles) published in the 1990–2025 period.

## Figures and Tables

**Table 1:** List of reported USP7 inhibitors and activators.

	Compound	Molecular Formula	Molecular Weigh	IC_50_ (USP7)	Preclinical Studies
Covalent Inhibitors	**HBX19818**	C_25_H_28_ClN_3_O	421.96	28.1 µM	Immunology, DUB selectivity, mice tumor models, TAM targeting [188].
	**HBX28258**	C_26_H_30_ClN_3_O	435.99	22.6 µM	Immunology, DUB selectivity, mice tumor models, TAM targeting [188].
	**P5091**	C_12_H_7_Cl_2_NO_3_S_2_	348.22	4.2 µM	Tumor cell lines, xenograft, combination therapies [18].
	**P22077**	C_12_H_7_F_2_NO_3_S_2_	315.32	8.0 µM	DUB selectivity, preliminary in vivo evaluation, activity-based protein profiling [73].
	**P50429**	C_18_H_11_Cl_2_N_3_O_3_S_3_	484.39	0.42 µM	DUB selectivity, preliminary in vivo evaluation [178].
	**P217564**	C_16_H_12_Cl_2_N_2_O_5_S_2_	447.30	0.48 µM	DUB selectivity, preliminary in vivo evaluation [181].
	**C7**	C_10_H_5_Cl_2_N_3_O_3_S_2_	350.19	0.67 µM	**/**
	**C19**	C_16_H_9_Cl_2_FN_4_O_3_S_2_	459.29	1.35 µM	**/**
Noncovalent allosteric inhibitors	**L55**	C_28_H_31_ClN_6_O_4_	551.04	40.8 nM	**/**
	**FT671**	C_24_H_23_F_4_N_7_O_3_	533.48	52.0 nM	Tumor cell lines, MM1.S xenograft, DUB selectivity [182].
	**YCH2823**	C_29_H_32_N_4_O_4_	500.59	49.6 nM	**/**
	**XL188**	C_32_H_42_N_6_O_4_	574.71	90 nM	Tumor cell lines, mice tumor models, p53/MDM2 pathway regulation, DUB selectivity [184].
	**1**	C_22_H_24_BrN_3_O_3_S	490.42	0.30 µM	**/**
	**2a**	C_29_H_34_N_6_O_3_	514.63	0.25 µM	**/**
	**2b**	C_29_H_31_F_3_N_6_O_3_	568.60	22.00 nM	**/**
	**GNE-6640**	C_20_H_18_N_4_O	330.38	0.75 µM	Triple-negative breast cancer, DUB selectivity, p53/MDM2 pathway regulation, combination therapies [171].
	**GNE-6776**	C_20_H_20_N_4_O_2_	348.40	1.35 µM	Triple-negative breast cancer, DUB selectivity, in vivo tumor models, combination therapies [171].
	**USP7-797**	C_27_H_28_ClN_3_O_3_S	510.05	0.50 nM	Tumor cell lines, DUB selectivity, V517F resistance models, in vivo tumor models [187].
Covalent allosteric inhibitors	**HBX41108**	C_13_H_3_ClN_4_O	266.64	0.42 µM	Tumor cell lines, p53 stabilization, p53 target activation, apoptosis [188].
	**XL177A**	C_48_H_57_ClN_8_O_5_	861.47	0.34 nM	Large tumor cell panel, xenograft, activity-based protein profiling [34].
	**FT827**	C_27_H_28_N_6_O_5_S	548.61	52.00 nM	Tumor cell lines, crystallography, activity-based protein profiling [182].
PROTACs	**PROTAC 17**	C_50_H_57_N_9_O_9_	928.06	1.60 µM	**/**
	**U7D-1**	C_53_H_65_N_9_O_7_	940.16	6.00 nM	**/**
Natural inhibitors	**Spongiacidin A**	C_11_H_11_Br_2_N_5_O_2_	405.05	8.50 µM	**/**
	**Spongiacidin C**	C_11_H_10_N_4_O_3_	246.23	3.80 µM	**/**
	**Xestoquinone**	C_20_H_14_O_4_	318.32	0.13 µM	**/**
	**Sulawesin A**	C_25_H_30_O_6_	426.20	2.8 µM	**/**
USP7 activators	**Asthemizole**	C_28_H_31_FN_4_O	458.57	**/**	**/**
	**Sertraline**	C_17_H_17_Cl_2_N	306.22	**/**	**/**
	**MS-8**	C_22_H_32_N_4_O_3_	400.52	**/**	**/**

## Data Availability

No new data were created or analyzed in this study. Data sharing is not applicable to this article.

## Abbreviations

The following abbreviations are used in this manuscript:

53BP1	p53-binding protein 1
ABCB1	ATP-binding cassette sub-family B member 1
Akt	Protein kinase B
AML	Acute myelogenous leukemia
ANXA1	Annexin A1
APC	Adenomatous polyposis coli
ARF	Alternate Reading Frame
ATR-Chk1	Ataxia Telangiectasia and Rad3-related protein Checkpoint kinase-1
BER	Base excision repair
BUB3	BUB3 Mitotic Checkpoint Protein
CARM1	Coactivator Associated Arginine Methyltransferase 1
CBP	CREB-binding protein
CCDC6	Coiled-coil domain-containing protein 6
CCF	Cytoplasmic chromatin fragments
CDC25A	Cell Division Cycle 25A
cGAS-STING	Cyclic GMP-AMP synthase-stimulator of interferon genes
CID	Catenin inhibitory domain
CK1α	Casein kinase 1α
CLL	Chronic lymphoid leukemia
CRBN	Cereblon
CRC	Colorectal cancer
DAXX	Death domain-associated protein
DDX3X	DEAD-box helicase 3 X-linked
DNMT1	DNA methyltransferase-1
DNMT1	DNA methyltransferase 1
DSB	Double Strand Break
DUB	Deubiquitinate
DUBTACs	DUB Targeting Chimeras
EBNA1	Epstein–Barr nuclear antigen 1
ELK4	ETS-like transcription factor 4
EMT	Epithelial–mesenchymal transition
ER	Estrogen receptor
ERK	Extracellular signal-regulated kinase
ETS	Electron transport system
EZH2	Zeste homolog 2 enhancer
FOXO1	Forkhead box protein O1
FoxO4	Forkhead box O4
FOXO6	Forkhead box protein O7
FOXP3	Forkhead box P3
GG-NER	Global genome NER
GLUT1	Glucose transporter 1
Grb2	Growth factor receptor-bound protein 2
GSK3β	Glycogen synthase kinase 3β
H3K4me1/2	Histone H3 at lysine 4
H3K9me1/2	Histone H3 at lysine 9
HAFOUS	Hao–Fountain Syndrome
HAUSP	Herpesvirus-associated ubiquitin-specific protease
HCC	Hepatocellular carcinoma
HDAC1	Histone deacetylase 1
HER2	Human epidermal growth factor receptor
HIF-1α	Hypoxia-inducible factor 1-alpha
HIFs	Hypoxia-inducible factors
hnRNPA1	Heterogeneous Nuclear Ribonucleoprotein A1
HNSSC	Head and neck squamous cell carcinoma
HR	Homologous recombination
ICP0	Infected cellular polypeptide 0
IFNGR1	Interferon gamma receptor 1
IGF	Insulin/insulin-like growth factor
IL-6	Interleukin 6
IRF-4	Viral interferon regulatory factor 4
IRS	Insulin receptor substrate
IκBs	NF-κB inhibitors
JAMM	JAMM/MPN domain-associated metallopeptidase
JMJD3	Jumonji domain-containing protein-3
K63	Lysine residue
LANA	Latency-associated nuclear antigen
LSD1	Lysine-specific histone demethylase 1
HLSD1	Histone lysine demethylase 1
Maged1	Melanoma-associated antigen D1
MCPIP	Monocyte chemotactic protein-induced proteins
MDC1	Mediator of DNA damage checkpoint protein 1
MDM2	Murine double minute 2
MDMX	Murine double minute X
MIG-6	Mitogen-inducible gene-6
MINDY	MIU-containing novel DUB family
MJD	Machado–Joseph disease protein domain proteases
MM1.S	Multiple myeloma
MMR	Mismatch repair
MRN	MRE11-RAD50-NBS1
NB	Neuroblastoma
NEK2	NIMA-related kinase 2
NER	Nucleotide excision repair
NF-κB	Nuclear factor-kappa B
NHEJ	Non-homologous end joining
NOTCH	Notch signaling pathway
NPM1	Nucleophosmin 1
NSCLC	Non-small cell lung cancer
OTU	Otubain domain ubiquitin-binding proteins
PD-1	Programmed cell death 1
PD-L1	Programmed cell death ligand 1
PI3K	Phosphatidylinositol 3-kinase
PLK1	Polo-like kinase 1
PPPDE	Permuted papain fold peptidase of dsDNA viruses and eukaryotes
PROTAC	Proteolysis-targeting chimera
PTEN	Phosphatase and tensin homolog
PVT	Paraventricular thalamus
RAP80	Receptor-associated protein 80
ROS	Reactive oxygen species
SIRT7	Sirtuin 7
SMAD	Small mother against decapentaplegic
TC-NER	Transcription coupled NER
Teff	Effector T cells
TNBC	Triple-negative breast cancer
TNF	Tumor necrosis factor
TPD	Targeted protein degradation
TRAF	Tumor necrosis receptor-associated factor
Treg	Regulatory T cells
TRIM27	Tripartite Motif Containing 27
TRIP13	Thyroid hormone receptor interactor 13
TSP-1	Thrombospondin-1
TSPYL5	Testis-specific protein Y-encoded-like 5
UBL1-5	Ubiquitin-like domains 1-5
UCH	Ubiquitin C-terminal hydrolases
UHRF1	Ubiquitin-like with PHD and ring finger domain 1
UPS	Ubiquitin–proteasome system
USP	Ubiquitin-specific proteases
USP7	Ubiquitin-specific proteases 7
USP7S	Ser18-containing isoform of USP7
VEGF	Vascular endothelial growth factor
ZUFSP	Zinc finger with UFM1-specific peptidase domain protein
βTrCp	β-transducin repeat-containing protein
